# Factor X deficiency: An uncommon presentation of AL amyloidosis

**DOI:** 10.3109/03009734.2012.690457

**Published:** 2012-10-30

**Authors:** Ajaydas T. Manikkan

**Affiliations:** Emory University Hospital, 1364 Clifton Road NE, Atlanta, GA 30322, USA

**Keywords:** AL amyloidosis, blood coagulation disorders, factor X deficiency

## Abstract

Factor X deficiency is the most common coagulation factor deficiency amongst patients with AL amyloidosis. It presumably occurs due to adsorption of factor X to amyloid fibrils. The deficiency of this factor, in conjunction with other hemostatic defects, can cause bleeding complications. A case of acquired factor X deficiency due to AL amyloidosis is reported, where abnormal coagulation parameters were the only presenting feature.

## Introduction

AL amyloidosis can be associated with several hemostatic defects, including deficiency of one or more coagulation factors. These hemostatic defects can cause bleeding complications and are often the presenting manifestation of the disease. A case of AL amyloidosis is presented, where abnormal coagulation parameters were the only presenting sign of the disease.

## Case report

A 74-year-old male presented with a fracture of the right femoral head and shaft after an accidental fall. Routine blood work in the emergency room revealed abnormal coagulation parameters. The patient was admitted to the medicine service for further evaluation and management of the coagulopathy in anticipation of hip surgery.

On admission, the patient only complained of right hip pain. He denied bleeding symptoms, fever, chills, night sweats, or weight loss. The patient had undergone hemorrhoidectomy 15 years back without bleeding complications. He denied alcohol, tobacco, or drug use. There was no family history of abnormal bleeding. He was not taking anti-platelet or anticoagulant medications.

At presentation, his vital signs were normal. Examination of the oral cavity did not show any petechiae or gingival anomalies. Cardiovascular and pulmonary examinations were unremarkable. There was no abdominal organomegaly. Musculoskeletal examination showed limited range of motion of the right hip but no hematoma formation. Skin examination showed bruises on the right arm. Neurological examination was non-focal. There was no lymphadenopathy.

Laboratory evaluation revealed hemoglobin of 10.8 g/dL (normal range 12.9–16.1 g/dL) and hematocrit of 31.8% (normal 37.7%–46.5%). Serum electrolytes and liver enzymes were normal. Total protein levels were in the normal range. Urinalysis was unremarkable. His prothrombin time (PT) was 32.7 s (normal 10.8–13.1 s), partial thromboplastin time (PTT) was 67.7 s (normal 25.0–39.0 s), and international normalized ratio was 2.48 (normal 0.9–1.2). D-dimer level was elevated, but fibrinogen level was not decreased, ruling out disseminated intravascular coagulation. Thrombin time was within normal limits. PT and PTT mixing studies were done, and they did not correct to normal. The lupus-sensitive PTT was prolonged and confirmed by a positive phospholipid neutralization procedure, suggesting presence of lupus anticoagulant activity. However, the dilute Russell's viper venom time was within normal limits. In addition, the ELISAs for anti-beta2-glycoprotein 1 and anti-cardiolipin antibodies were within normal limits. Coagulation factor levels were checked, and all except factor X levels were within normal limits. Factor X level was extremely low at 5%.

The factor X deficiency was considered to be acquired given the lack of personal or family history of bleeding. Infusions of fresh frozen plasma did not correct the factor X levels, raising suspicion of amyloidosis. Work-up for a gammopathy was undertaken. Neither serum protein electrophoresis nor urine protein electrophoresis revealed paraproteins. Immunoglobulin G, M, and A levels were within normal limits. However, free lambda levels were elevated at 39.10 mg/L (normal 5.71–26.30 mg/L). Free kappa levels were normal. Beta2 microglobulin was mildly elevated at 3.79 mg/L (normal 0.70–3.40 mg/L).

The patient received two transfusions of factor eight inhibitor bypass activity (FEIBA) anti-inhibitor coagulation complex and subsequently underwent a right hemiarthroplasty without any bleeding complications. A touch preparation of the femoral neck showed increased plasma cells comprising approximately 15%–20% of the nucleated cells. A Congo red stain demonstrated Congophilic material that displayed apple-green birefringence on polarization. Flow cytometric immunophenotyping of the femur demonstrated the presence of a clonal population of cells which were consistent with plasma cells. The cells were CD38+/CD45- and exhibited lambda light chain restriction. The patient underwent further evaluation for amyloidosis including an abdominal fat pad biopsy and a cardiac MRI. The fat pad biopsy was Congo red stain-negative, but the cardiac MRI showed mild left ventricular hypertrophy and delayed enhancement pattern consistent with cardiac amyloidosis. The patient was diagnosed with acquired factor X deficiency secondary to AL amyloidosis and discharged to follow up with hematology.

The patient was treated with a combination of melphalan and dexamethasone. However, he failed to respond after six cycles, with no significant improvement in his factor X levels. He continues to receive chemotherapy with a regimen consisting of melphalan, dexamethasone, and bortezomib.

## Discussion

Factor X deficiency is the most common coagulation factor deficiency in patients with AL amyloidosis (1). It has been reported to occur in 8.7% to 14% of patients (1,2). The half-life of factor X is shortened due to its adsorption to amyloid fibrils, explaining the deficiency (3). Hepatic synthetic defect or urinary protein losses due to nephrotic syndrome are not causes of the factor X deficiency (1).

Although factor X deficiency is the most common factor deficiency in AL amyloidosis, it is uncommon for it to be the presenting feature of the disease. In the study by Mumford et al., only 3 of the 337 (0.9%) enrolled patients presented after incidental discovery of abnormal coagulation parameters (2). In fact, the authors of another large series of 368 patients suggest that patients with AL amyloidosis be screened for factor X deficiency (1).

Factor X is a coagulation factor involved in both the intrinsic and extrinsic pathways, explaining the prolongation of PT and PTT in these patients. However, factor X levels do not correlate with the magnitude of prolongation of these coagulation parameters (1,2). Additionally, in factor X deficiency due to AL amyloidosis, the factor levels may not be the major determinant of bleeding complications or their severity, since these patients may have other hemostatic defects (2,4). These include small-vessel fragility due to amyloid infiltration, deficiencies of other coagulation factors, abnormal fibrin polymerization, vitamin K deficiency, thrombocytopenia, and dysfunctional platelets (2,4-7).

Treatment of factor X deficiency associated with AL amyloidosis is difficult. Evidence is sparse to guide management of bleeding episodes, and most of the information available is in the form of case reports. Replacement with prothrombin complexes and plasma may not be yielding as the factor X in these products would get adsorbed onto the amyloid and, thus, be removed from the circulation quickly (8). Recombinant factor VIIa and plasma exchange have been reported to be successful (8,9). By removing a considerable burden of amyloid, splenectomy has been shown to improve the coagulopathy (10). Finally, treating the underlying disease with chemotherapy and stem cell transplantation can lead to improvement in factor X levels (1).
